# Politics and confidence toward the COVID-19 vaccination: A Brazilian cross-sectional study

**DOI:** 10.1080/21645515.2024.2318139

**Published:** 2024-02-26

**Authors:** Marco Antonio Catussi Paschoalotto, Joana Cima, Eduardo Costa, Sara Valente de Almeida, Joana Gomes da Costa, João Vasco Santos, Claudia Souza Passador, João Luiz Passador, Pedro Pita Barros

**Affiliations:** a School of Economics and Management, University of Minho, Braga, Portugal; b Research Center in Political Science (CICP), University of Minho, Braga, Portugal; c Centre for Research in Economics and Management (NIPE), University of Minho, Braga, Portugal; d Nova School of Business and Economics, Nova University of Lisbon, Lisbon, Portugal; e School of Public Health, Imperial College London, London, UK; f Center for Economics and Finance; School of Economics and Management, University of Porto, Porto, Portugal; g MEDCIDS – Department of Community Medicine, Information and Health Decision Sciences, Faculty of Medicine, University of Porto, Porto, Portugal; h CINTESIS – Center for Health Technology and Services Research, Faculty of Medicine, University of Porto, Porto, Portugal; i Public Health Unit, ACES Grande Porto VIII - Espinho/Gaia, ARS Norte, Porto, Portugal; j School of Economics, Business Administration and Accounting at Ribeirão Preto, University of São Paulo, Ribeirão Preto, Brazil

**Keywords:** COVID-19, vaccines, pandemic, politics, vaccination hesitance

## Abstract

This study has the aim of assessing the Brazilian perceptions, influencing factors and political positioning on the confidence concerning COVID-19 vaccination. To achieve the objective, the methods rely on a cross-sectional survey of Brazilian citizens, distributed through different social networks. The sample is composed of 1,670 valid responses, collected from almost all Brazilian states and state capitals. To analyze the data and give a clear view of the variables’ relationship, the study used bivariate and comparative graphs. Results show a higher level of confidence in vaccines from Pfizer and AstraZeneca, while the lower level of confidence is associated with vaccines from Sinopharm and Sputinik5. Vaccine efficacy is the most significant influencing factor that helps in the decision to get vaccinated. Also, individuals are less willing to get vaccinated if their political preferences are related to the right-wing. The results led to three main health and social implications: i) the vaccination strategy campaigns should take in count vaccine efficacy and political aspects; ii) the vaccination process should be adapted to regions with different political positions; and iii) a reinforcement in the educational policies of the vaccine’s importance to the public health, to avoid the politization of a health issue.

## Introduction

The COVID-19 pandemic and its high mortality rate^1^ brought back to the public health and modern society debate the factors that could influence the willingness of an individual to take or not the vaccine shot.^2–4^ Different research over all the world have been developed to understand this phenomenon: a) low- and middle-income countries with high level of confidence in the COVID-19 vaccines,^5–7^ and higher than USA and Russia;^8;^ b) social media, political engagement and trust in the institutions being significative to explain vaccine hesitancy,^9,10^ and c) sociodemographic characteristics and political polarization influence the vaccine’s confidence.^11,12^

Brazil has become one of the main epicenters of COVID-19 cases and deaths globally, with uneven contamination among the states and lacking a unified epidemiological strategy and rapid vaccination.^13^ Previous studies in Brazil have indicated the association between political ideology and willingness to take the COVID-19,^9,14^ negatively affecting the need for fast vaccination from the Brazilian population.^15^ In the Brazilian case of the vaccination process, understanding the variables that influence confidence in the predisposition to take vaccines is fundamental.^16^ Therefore, among these factors, political positioning^16–19^ and characteristics of the available vaccines (country of origin, developing company, speed, lack of information, and efficacy)^16–18^ stand out.

To understand the above-described phenomenon, this study aims to analyze the relationship between political positioning and the presidential voting in the 2018 elections with the predisposition to take the COVID-19 vaccine, confidence in the available vaccines, and the degree of influence of the characteristics that make up the vaccines. This study is justified by the need to understand the perception of the Brazilian population regarding the COVID-19 vaccine, as well as to assist decision-makers in choosing vaccines and their communication.

## Material and methods

### Sample and data

To achieve the study’s aim, an online survey was conducted with citizens residing in Brazil between November 25, 2020, and January 11, 2021, through social networks Facebook, Instagram, Email, and Whatsapp. The online survey was based on the Qualtrics software to facilitate the data collection, management, and analysis.

The collected sample has 1,670 valid responses, being a non-representative sample but with sociodemographic characteristics close to the Brazilian population. Specifically, the sample includes records from 24 states and 263 participating municipalities. 61.7% of the sample is composed of women, and there is a concentration of responses in the age group of 19 to 64 years, residing in urban areas, and with families of 3 to 4 members.^20^

### Measures of variables

The developed questionnaire was approved by the Scientific Ethics Committee of the School of Business and Economics at NOVA University of Lisbon (Portugal) on November 23, 2020, and includes, among others, the following questions:

1. In the presence of a COVID-19 vaccine, would you be willing to take it? Possible answers: Yes, No, and Maybe.
2. What is your degree of confidence in the following vaccines that may be available in Brazil? A) Oxford/AstraZeneca (United Kingdom), B) Sinovac/Butantan (China), C) Pfizer (United States and Germany), D) Moderna (United States), E) Sinopharm (China), F) Sputnik5 (Russia), G) Covaxx and Novavax (United States), and H) Janssen (Belgium). Possible answers: 1 - None, 2 - Low, 3 - Moderate, 4 - High, and 5 - Very High.
3. Regarding the factors listed below, what is the degree of influence in the decision to “take” or “not take” the COVID-19 vaccine? A) Country of origin of its development, B) Research institute or Developing company, C) Speed of vaccine production, D) Speed of vaccine testing, E) Lack of information in the production and testing process, F) Vaccine efficacy (vaccine immunization percentage). Possible answers: 1 - None, 2 - Low, 3 - Moderate, 4 - High, and 5 - Very High.
4. On a scale of 1 to 7, indicate the number that most closely resembles your political positioning. Possible answers (listed from left to right): 1 - Far Left, 2, 3, 4 - Center, 5, 6, 7 - Far Right.
5. In the last presidential election (2018), did you vote for the president who eventually won that election?. Possible answers: Yes and No.

Table 1 presents the answers distribution of each question. The main characteristics are: 1) All the vaccines have at least 50% of the sample higher than Moderate in the level of confidence; 2) All the influence factors have at least 50% of the sample higher than Moderate to take or not to take the COVID-19 vaccine; 3) 29% of the sample would not be or maybe would not be willing to take the COVID-19 vaccine; 4) 69.5% of the sample are concentrated between center and center-left political positioning; and 5) 66.3% of the sample did not vote in the elected presidential in the 2018 Brazilian elections.

**Table 1.** Sample characteristics.

Questions	%				
**1) The level of confidence in the following vaccines:**	**1 - Null**	**2 - Low**	**3 - Moderate**	**4 - High**	**5 - Very High**
Oxford/Astrazeneca (UK)	2.2	7.9	27.9	36.9	25.1
Sinovac/Butantan (China)	10.4	15.3	22.7	27.3	24.3
Pfizer (USA and Germany)	2.6	8.1	28.6	33.9	26.8
Moderna (USA)	2.9	11.4	34.4	30.4	20.9
Sinopharm (China)	14.0	22.2	28.4	19.6	15.7
Sputinik5 (Russia)	10.4	27.0	32.5	19.0	11.1
Covaxx e Novavax (USA)	3.7	14.3	37.9	26.1	18.0
Janssen (Belgium)	4.5	16.0	37.1	24.6	17.7
**2) Degree of influence on the decision to take or not take the COVID-19 vaccine based on:**	**1 - Null**	**2 - Low**	**3 - Moderate**	**4 - High**	**5 - Very High**
Country of Origin	28.8	17.0	20.8	17.5	15.9
Research Institute or Enterprise of Origin	14.9	11.2	21.3	29.3	23.3
Vaccine Production speed	14.4	15.8	30.7	22.2	16.8
Vaccine tests speed	8.9	13.1	27.8	27.8	22.3
Lack of information about the vaccine	5.8	10.3	16.6	24.3	43.0
Vaccine efficacy (% of immunization)	3.5	7.4	17.0	27.1	45.0
**3) In the presence of a COVID-19 vaccine, would you be willing to take it?**	**Yes**	**No**	**Maybe**	**4) Political Positioning:**	
	70.9	7.8	21.2	1	2.9
				2	17.8
				3	24.6
**5) In the last presidential election (2018), did you vote for the president who eventually won that election?**	**Yes**	**No**		4	27.0
	33.7	66.3		5	14.7
				6	9.6
				7	3.4

### Data analysis procedure

For data analysis, the study used comparative graphs and descriptive statistics on the main variables: 1) predisposition to take or not take the vaccine, 2) degree of confidence in the possible vaccines available, 3) degree of influence of the characteristics linked to the vaccines, 4) political positioning, and 5) voting for the elected president in the last elections (October 2018).

## Results

The analysis is divided into 4 figures, divided in the sections A, B, C, and D.

**Figure 1.**
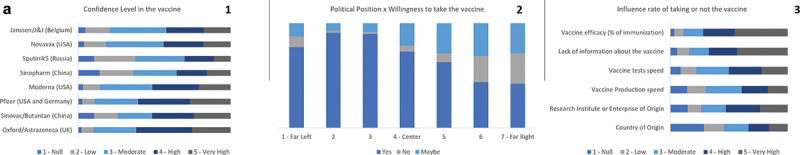
(a). Confidence level in the vaccine x political position x influence factors of taking or not the vaccine.

In line with graph 1 presented in the Figure 1, initially, greater confidence is observed in the Pfizer, Oxford/AstraZeneca, and Moderna vaccines (with higher levels of High and Very High), and less confidence in the Sputnik5, Sinopharm, and Sinovac/Butantan vaccines (with higher levels of Null and Low), compared to the other available vaccines. Looking at graph 3 in the same figure, it is noted that the main influencing factors in deciding to take or not take the vaccine are the vaccine’s efficacy (vaccine immunization percentage) and the lack of information in the production and testing process. On graph 2the relationship between political positioning and the decision to take or not take the vaccine shows a greater reluctance of those positioned as center-right or far-right (higher values of No and Maybe), going in the opposite direction to the positioning of center and center-left, who mostly intend to take the vaccine (higher values of Yes), with a small reduction in the far left.

- Higher level of confidence in the Pfizer and Oxford/AstraZeneca vaccines, while lower level of confidence in the Sputnik5 and Sinopharm;
- Vaccine efficacy and lack of information about the vaccine are the most influencers to take or not the vaccine;
- There is a crescent tendence of taking the vaccine from the far-right to the center-left political position.

**Figure 2.**
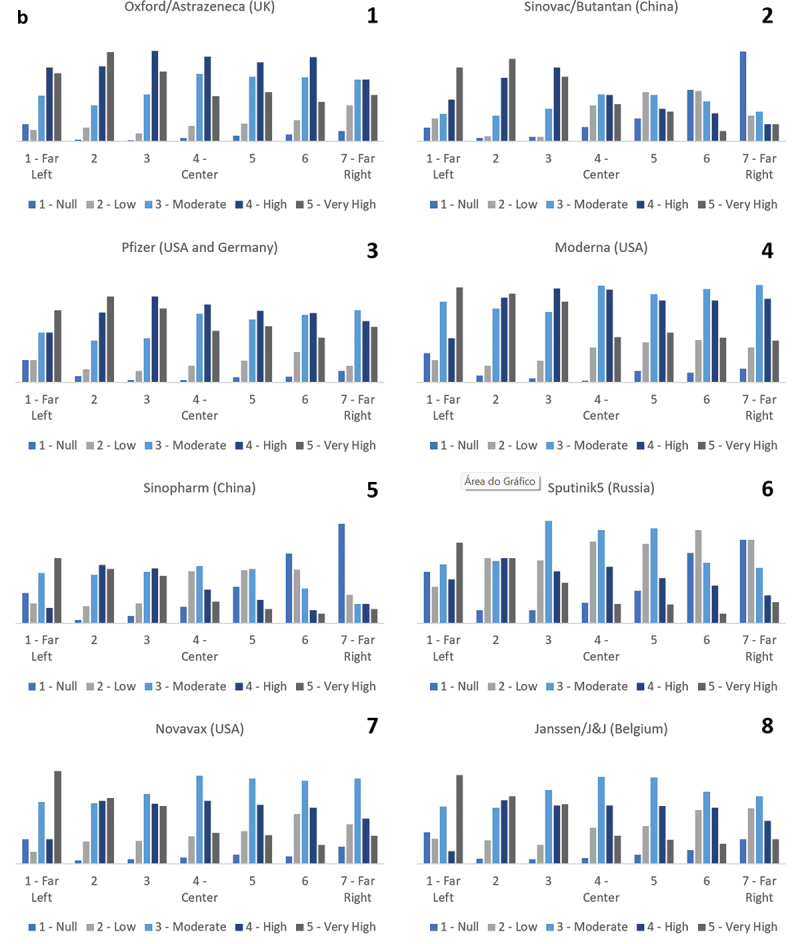
(b). Confidence level in the vaccines.

In the Figure 2, where we observe the relationship between political positioning and the degree of confidence in each of the analyzed vaccines, graphs 1 to 8 highlight a greater variation of confidence between political spectrums in the Sputnik5, Sinopharm, and Sinovac/Butantan vaccines, with greater distrust as one positions to the right (Null and Low) and confidence when positioned to the left (High and Very High).

- There is a high level of variation in vaccine’s confidence between right and left political positions over the Sputnik5, Sinopharm, and Sinovac/Butantan.

**Figure 3.**
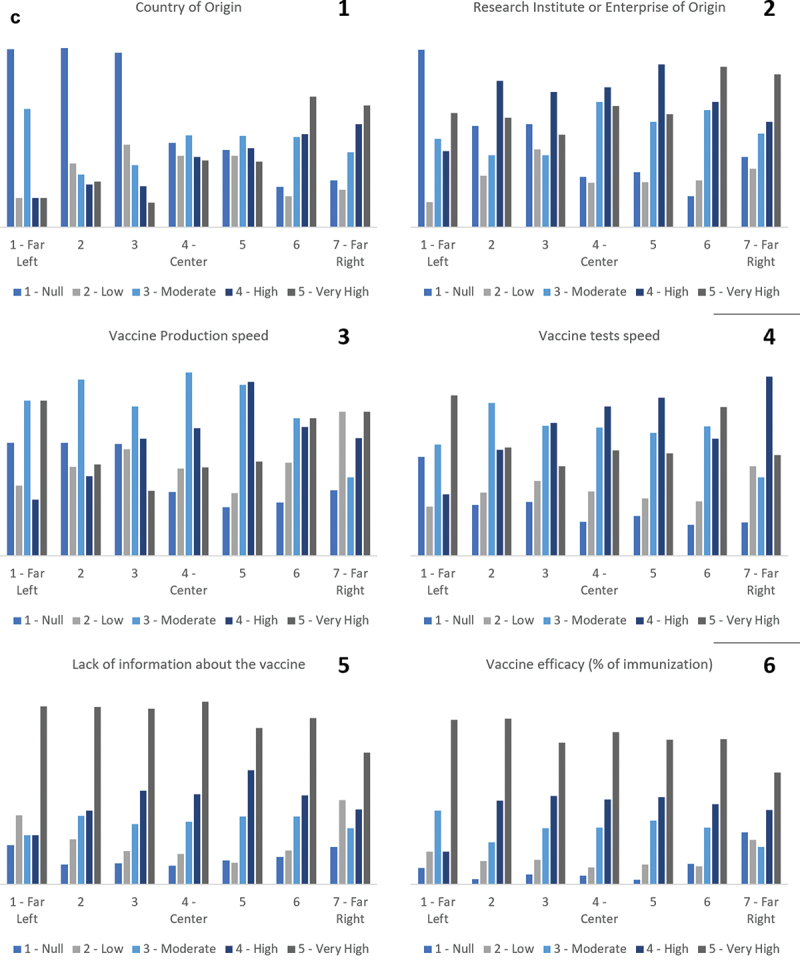
Influence factors of taking or not the vaccine.

The Figure 3 presents the degree of influence of the characteristics that make up the vaccine in the predisposition to take it or not with political positioning. In this figure, graphs 1 to 6 point out: I) a variation between political positions in the degree of influence of the characteristic “Country of origin of its development,” with the left-wing political spectra portraying a low degree of influence (higher values of Null and Low), while the far-right demonstrates a high degree of influence (higher values of High and Very High); II) a lack of standardization in the degree of influence in different political positions of the characteristics “Research institute or Entreprise of origin,” “Vaccine production sped” and “Vaccine testing speed”; and III) a strong influence of the characteristics “Lack of information in the production and testing process of the Vaccine” and “Efficacy of the Vaccine (vaccine immunization percentage)” in all analyzed political positions.

- The factor “Country of origin of its development” has a high level of variation between political positions, which to the far-right side plays an important role, while to the center and left side does not.

**Figure 4.**
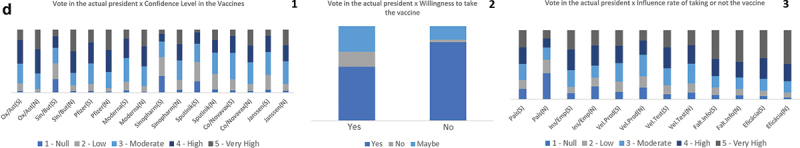
(d). Vote in the actual president in the last election x political position x influence factors of taking or not the vaccine.

The last figure, 4, reports the association between having voted in the last presidential election for the winning president and confidence in vaccines, the decision to take or not take the vaccine, and the degree of influence of the characteristics to take or not take the vaccine. Initially, graph 1 of Figure 4 describes a higher level of confidence in all analyzed vaccines from those who voted “No” for the winning president in the 2018 elections compared to those who voted “Yes.” In addition, there can be pointed out a greater distrust in the Sinovac/Butantan, Sinopharm, and Sputnik5 vaccines from those who voted “Yes” for the elected president in the last election (higher values of High and Very High). In graph 3 of Figure 4, there is a low disparity in the degree of influence of the listed characteristics between having voted or not for the elected president, with the exception of the characteristic “Country of origin of its development,” where those who voted “Yes” in the last election for the winning president describe a higher degree of influence compared to those who did not vote. Finally, when analyzing graph 2 in Figure 4, there is a lower willingness to take the COVID-19 vaccine from those who voted “Yes” for the elected president in 2018 (with higher values of “No” and “Maybe”), compared to those who did not vote for the last elected president.

- Those who voted “Yes” to the winning president in the 2018 Brazilian election have lower level of willingness to take the COVID-19 vaccines compared to those who voted “No;”
- The factor “Country of origin of its development” has a high level of influence on those who voted “Yes” to the winning president in the 2018 Brazilian election compared to those who voted “No.”

## Discussion

At the end of this work, the following discussions can be listed:

- those who voted for the current Brazilian president in the 2018 elections^17^ and those who positioned themselves politically further to the right^16,18,19^ are more reluctant to take the COVID-19 vaccine,^9,10^ have less confidence in the Sputinik5 (Russia), Sinopharm (China), and Sinovac/Butantan (China) vaccines, and indeed, consider that the country of origin of the vaccine development strongly affects their decision
- the characteristics that most influence the decision to take the vaccine are the lack of information about the production, testing, and efficacy (immunization percentage).^16,18^

Based on the presented results, this work confirmed previous studies that pointed out the influence of the political positioning in the COVID-19 vaccine confidence^11–12,16–18^ and willingness to take or not.^8–10^ On the other side, this paper pointed out two important findings: a) country of origin of the vaccine as a factor that influences right-wing individuals; and b) lack of information about the production and testing, and efficacy of the vaccines are the most influencers to take or not to take.

Thus, the need for a unified discourse for mass vaccination^13^ and based on science, combating the lack of information or fake news,^17^ is reiterated. Also, the strategy of the vaccination programs should pay attention on the political side of the population, due to its relationship with variables to take or not the vaccine, such as country of origin.

## Conclusion

Therefore, this paper advanced by analyzing the relationship of supporters of the elected president and political positioning with the decision to take or not take the vaccine, confidence in them, and possible influencing factors. Moreover, the results provide new insights of practical and social implications, such as:

1. Decision-makers should pay attention into the population characteristics when formulating the strategies to the vaccination program, to better achieve the results;
2. Invest in health education through of the street-level bureaucrats, to keep the society close to the scientific information;
3. Establish long-term strategies to deal with new outbreaks and hesitancy of following the public health measures, based and applied by the primary health care teams, such as the strategy family teams.

The limitations of the study surround a non-representative sample and the use of simple statistics. As future studies, it is proposed to replicate the survey at different times of the pandemic and in different types of outbreaks, and to deepen the debate with qualitative and comparative studies in different contexts
